# Automated Deep Learning Based Cardiac Quantification in Hypertrophic Cardiomyopathy: A Comparative Study with Manual Segmentation

**DOI:** 10.15388/Amed.2025.32.2.8

**Published:** 2025-12-30

**Authors:** Shivam Angiras, Deb Kumar Boruah, Pranjal Phukan, Kalyan Sarma, Prince Das, Rajeev Bharadwaj, Harshit Jain, Ajmal Roshan

**Affiliations:** 1 Department of Diagnostic and Interventional Radiology, All India Institute of Medical Sciences Guwahati, Assam, India; 2 Department of Diagnostic and Interventional Radiology, All India Institute of Medical Sciences Guwahati, Assam, India; 3 Department of Diagnostic and Interventional Radiology, All India Institute of Medical Sciences Guwahati, Assam, India; 4 Department of Diagnostic and Interventional Radiology, All India Institute of Medical Sciences Guwahati, Assam, India; 5 Department of Diagnostic and Interventional Radiology, All India Institute of Medical Sciences Guwahati, Assam, India; 6 Department of Cardiology, All India Institute of Medical Sciences Guwahati, Assam, India; 7 Department of Diagnostic and Interventional Radiology, All India Institute of Medical Sciences Guwahati, Assam, India; 8 Department of Diagnostic and Interventional Radiology, All India Institute of Medical Sciences Guwahati, Assam, India

**Keywords:** hypertrophic cardiomyopathy, deep learning, cardiac MRI, mitral regurgitation, automated segmentation, left ventricular function, hipertrofinė kardiomiopatija, gilusis mokymasis, širdies MRT, mitralinio vožtuvo nesandarumas, automatinis segmentavimas, kairiojo skilvelio funkcija

## Abstract

**Background:**

Hypertrophic CardioMyopathy (HCM) is the most prevalent inherited cardiac disorder, where accurate assessment of Left Ventricular (LV) function and Mitral Regurgitation (MR) is crucial. Cardiac Magnetic Resonance (CMR) imaging is considered the gold standard for evaluating these parameters. Recently, Deep Learning (DL) algorithms have emerged to automate cardiac quantification, but their performance in complex pathologies such as HCM still requires validation.

**Purpose:**

To compare the performance of a fully automated deep learning-based cardiac segmentation software (SW 2) (SuiteHEART) with conventional manual segmentation (SW 1) (syngo.Via) for quantifying crucial cardiac parameters in patients with HCM.

**Materials and Methods:**

In this prospective study, 25 consecutive adult patients (mean age 49±12 years) with HCM referred for CMR at our institute were included. CMR examinations were performed by using a 3.0 Tesla scanner (Siemens Vida). The key parameters assessed included Left Ventricular Ejection Fraction (LVEF), End-Diastolic Volume (LVEDV), Stroke Volume (LVSV), Aortic Forward Flow (AoF), Mitral Regurgitation (MR), and Pressure Gradient (PG) across the LVOT. Manual and automated segmentations were performed by using syngo.Via (SW 1) and SuiteHEART software (SW 2), respectively. Statistical analysis included paired t-tests, linear regression, and Bland–Altman analysis.

**Results:**

There was a strong correlation between DL-based and manual measurements for LVEF (r=0.91), LVEDV (r=0.89), LVSV (r=0.87), AoF (r=0.86), MR (r=0.84), and PG (r=0.81) (all *p*<0.001). Bland–Altman analysis demonstrated acceptable limits of agreement, with no significant bias. Automated segmentation significantly reduced post-processing time compared to manual methods (*p*<0.001).

**Conclusion:**

Fully automated DL-based cardiac quantification provides accurate and reproducible assessment of the LV function, MR, and flow parameters in HCM patients, closely matching manual segmentation results. Incorporation of DL algorithms can substantially streamline the clinical workflow, although careful validation remains necessary in structurally complex cases such as HCM.

## Abstract (it)

**Įvadas:**

Hipertrofinė kardiomiopatija (HCM) yra labiausiai paplitusi paveldima širdies liga, kuriai diagnozuoti labai svarbu tiksliai įvertinti kairiojo skilvelio (LV) funkciją ir mitralinio vožtuvo (MR) nesandarumą. Širdies magnetinio rezonanso (CMR) vaizdinimas laikomas šių parametrų vertinimo aukščiausiu standartu. Neseniai atsirado giliojo mokymosi (DL) algoritmai, skirti širdies kiekybiniam vertinimui automatizuoti, tačiau jų veiksmingumas sudėtingų patologijų, tokių kaip HCM, atveju turi būti patvirtintas.

**Medžiagos ir metodai:**

Šiame prospektyviniame tyrime dalyvavo 25 suaugę pacientai (vidutinis amžius 49±12 metų) su HCM, kurie siųsti į mūsų institutą CMR tyrimui. Širdies magnetinio rezonanso tyrimai buvo atliekami naudojant 3,0 Tesla skenerį (Siemens Vida)). Vertinti pagrindiniai parametrai: kairiojo skilvelio išstūmimo frakcija (LVEF), diastolinis tūris (LVEDV), smūginis tūris (LVSV), aortos priekio srautas (AoF), mitralinio vožtuvo nesandarumas (MR) ir slėgio gradientas (PG) per LVOT. Rankinis ir automatinis segmentavimas buvo atliekamas naudojant atitinkamai syngo.Via (SW1) ir SuiteHEART (SW2) programinę įrangą. Statistinė analizė apėmė porinius t testus, linijinę regresiją ir Bland-Altman analizę.

**Rezultatai:**

Buvo nustatyta stipri DL pagrįstų ir rankinių matavimų LVEF (r = 0,91), LVEDV (r = 0,89), LVSV (r = 0,87), AoF (r = 0,86), MR (r = 0,84) ir PG (r = 0,81) (visi p < 0,001) koreliacija. Bland-Altman analizė parodė priimtinas sutapties ribas be reikšmingo nuokrypio. Automatinis segmentavimas gerokai sumažino apdorojimo laiką, palyginti su rankiniais matavimo metodais (p < 0,001).

**Išvados:**

Visiškai automatizuotas DL pagrįstas širdies kiekybinis vertinimas užtikrina tikslų ir pakartotinį HCM pacientų LV funkcijos, MR ir srauto parametrų vertinimą, kuris labai atitinka rankinio segmentavimo rezultatus. DL algoritmų įtraukimas gali itin supaprastinti klinikinio darbo srautą, tačiau sudėtingų struktūrinių atvejų, pvz., HCM, atveju vis dar būtina atidžiai atlikti patvirtinimą.

## Introduction

*Hypertrophic CardioMyopathy* (HCM) is the most common inherited cardiac disorder, affecting approximately 1 in 500 individuals [1–4]. *Magnetic Resonance Imaging* (MRI) plays a pivotal role in its evaluation, particularly for *Mitral Regurgitation* (MR) and myocardial tissue characterization [5]. In nearly 70% of HCM cases, *Left Ventricular Outflow Tract* (LVOT) obstruction arises from septal thickening and elevated pressure gradients above the *Aortic Valve* (AV), creating drag forces that draw the *Anterior Mitral Leaflet* (AML) into the LVOT, leading to ‘*Systolic Anterior Motion* (SAM)-dependent’ MR [6]. In 10–20% of cases, MR occurs independently of SAM, due to intrinsic mitral valve abnormalities [6–8]. While echocardiography is the first-line imaging tool, cardiac MRI offers more accurate assessment of mitral valve structure and regurgitant severity. The preferred MRI method uses 2D cine imaging and phase-contrast velocity mapping to calculate the *Mitral Regurgitant Volume* (MRV) [9].

Cardiac MRI is inherently time-intensive, both in acquisition and interpretation. Post-processing tasks like *Ejection Fraction* (LVEF) calculation require meticulous manual effort. Deep learning has significantly improved the workflow efficiency by enabling near-instantaneous segmentation, thereby allowing radiologists to validate outputs within minutes. This automation reduces manual workload and supports a broader use of MRI in centers with limited staffing or a high patient throughput.

*Cardiac Magnetic Resonance* (CMR) is the gold standard for assessing LVEF, *Left Ventricular Mass* (LVM), and *Right Ventricular Ejection Fraction* (RVEF) due to its high spatial resolution and independence from geometric assumptions [10]. It also provides superior reproducibility for quantifying the stroke volume and valvular regurgitations compared to echocardiography [11]. These advantages make CMR highly compatible with *Deep Learning* (DL) applications which offer an enhanced segmentation accuracy along with a reduced processing time [12].

However, in HCM, features like asymmetric hypertrophy, abnormal papillary muscles, or mid-cavity obstruction can hinder automated delineation of myocardial boundaries. These structural complexities may challenge DL-based calculations of LVEDV, stroke volume, and MR. Additionally, most AI-based tools focus on aortic and pulmonary flow, while LVOT pressure gradients – which are crucial in HCM – may be overlooked [12].

Given that algorithmic details are often proprietary, and that the impact of manual contour adjustments is uncertain, this study investigates the utility of commercially available DL-based segmentation software at our center. We compare its performance to manual segmentation in evaluating ventricular volumes, flow dynamics, LVOT gradients, and MR in patients with HCM.

## Materials and methods

We identified 25 consecutive patients (age 51±10years) with HCM referred for Cardiac MRI from the department of Cardiology at our institute (All India Institute of Medical Sciences, Guwahati). All patients were referred for the diagnosis or evaluation of severity of HCM on MRI. Patient demographics and clinical history were noted (Table 1).

**Table 1:** Patient demographics with sample size of 25 (male and female)

Sr. No.	Parameter	Value
1	Gender (M/F)	16/9
2	Age	49±12
3	Height (cm)	143±14
4	Weight	57±16
5	BSA	1.9±0.7
6	Heart rate	80±13

- Inclusion Criteria
  1. Adult patients (≥18 years) diagnosed with *Hypertrophic Cardiomyopathy (HCM)* as per 2020 ESC Guidelines including patients with septal reduction therapy (surgical myectomy or alcohol septal ablation);
  2. Referred for cardiac MRI at AIIMS Guwahati for confirmation of HCM diagnosis, severity of LV hypertrophy, evaluation of LGE or MR, LVOT;
  3. Patients providing written informed consent.
- Exclusion Criteria
  1. Incomplete or non-diagnostic quality MRI images;
  2. Known congenital heart disease or infiltrative cardiomyopathies (e.g., amyloidosis);
  3. Contraindications to MRI: ferromagnetic implant or claustrophobic patient;
  4. Poor breath-hold capacity affecting the image quality.
- MRI Parameters
  Performed on the *3.0 Tesla Siemens Vida* system with an 18-channel cardiac coil. TrueFISP pulse sequence used for cine imaging in all planes, i.e., 2 Chamber, 4 Chamber and short axis with a slice thickness of 7mm with a 2mm gap. The phase contrast sequence was planned at the level of aorta (1–1.5 cm above the aortic valve) and LVOT. Gadolinium-based contrast agent (0.1 mmol/Kg of patient) was used, and first pass perfusion, EGE (*Early Gadolinium Enhancement*) and LGE (*Late Gadolinium Enhancement*) sequences were acquired.
- AI Tools and Licensing
**Software 1: Manual Segmentation**
  - **Name:** syngo.Via (Siemens Healthineers);
  - **Modules Used:** Cardiac Function, Flow Quantification;
  - **Version:** syngo.Via VB60.
**Software 2: Deep Learning-based Segmentation**
  - **Name:** SuiteHEART (NeoSoft LLC, USA);
  - **Method:** Fully automated CNN-based segmentation of LV/RV and flow;
  - **Regulatory Status:** FDA 510(k) cleared (K203127);
  - **License Type:** Commercial institutional license; includes virtual training by vendor;
  - **Version Used:** SuiteHEART v5.4.
- Statistical Analysis

*IBM SPSS Statistics* was used for statistical analysis. *GraphPad Prism Version 9.5.1* was used for data visualization

1. Descriptive Statistics: Mean ± SD;
2. Paired t-tests: Comparison of DL vs manual parameters (EF, LVEDV, LVSV, MR, AF, PG);
3. Linear Regression Analysis: Agreement between DL and the manual method;
4. Bland–Altman Analysis (optional): Assessment of bias and limits of agreement;
5. *p*-value < 0.05 was considered statistically significant.

### 1. Image Acquisition

MRI was done on a *3T Siemens Vida* scanner with an 18-channel cardiac coil. A retrospectively gated *TrueFISP* sequence (TR 2.7 ms, TE 1.5 ms, flip angle 60°, temporal resolution 50 ms) was used during 8–10 second breath-holds to acquire standard long-axis (2-, 3-, and 4-chamber) and 6–8 short-axis slices (7 mm thickness, 2 mm gap) from base to apex. First-pass perfusion followed a gadolinium injection (0.1 mmol/kg), whereas late gadolinium enhancement was assessed by using PSIR. Flow quantification was performed by using 2D phase contrast imaging. The study was approved by the Institutional Review Board, and informed consent was obtained.

### 2. Clinical image analysis with manual method (SW 1)

**Figure 1:**
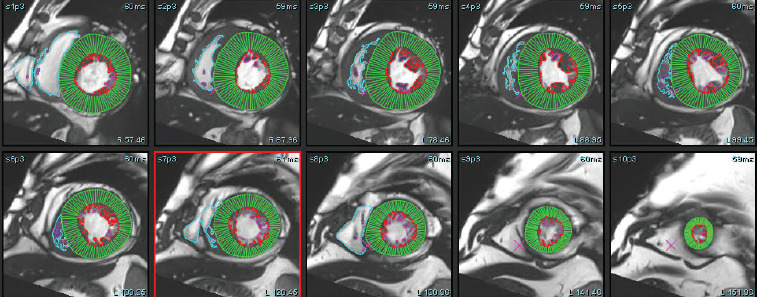
Deep learning-based automated segmentation of the left ventricle on short-axis cine CMR images. The software accurately identifies both epicardial and endocardial contours without user input, explicitly excluding papillary muscles from the myocardial mass. This fully automated contouring enables rapid and reproducible quantification of the left ventricular volumes and function. The displayed image represents the basal to apical segment slice analyzed by using DL-based post-processing software (SW 2).

**Figure 2:**
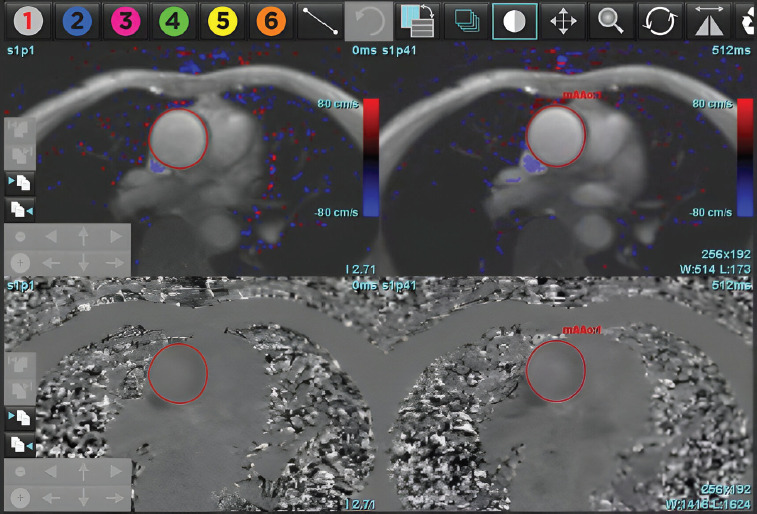
Automated identification of the ascending aorta and dynamic contour generation across all phases of the cardiac cycle using deep learning-based CMR post-processing software. The algorithm automatically detects the aortic lumen and applies phase-resolved contouring without any manual input, thereby enabling precise measurement of the aortic forward flow throughout systole and diastole. This automation facilitates efficient and reproducible flow quantification in the clinical practice.

The cine-CMR images were analyzed during routine clinical workflow by using commercial software (syngo.Via). Using the short-axis cine images, the LV and RV end-diastolic and end-systolic frames were identified. In each short-axis slice, the endocardial boundary of the LV and RV were manually delineated. LV and RV papillary muscles and trabecular tissue were included in the blood pool volume. In the LV basal slices, the LV contour was drawn to include the LV outflow tract to the level of the aortic valve cusps (see Figure 1). Contours for the postprocessing of flow study at the level of ascending aorta were drawn by using the manual method, whereas the DL method automatically recognized the aorta, and a contour was formed for all phases (see Figure 2). Simpson’s method of disks was used to calculate the LV Ejection fraction, as well as end-diastolic and end-systolic volumes (EF, EDV and ESV). These values were then reported and recorded. We divided the patient cohort into three clinically relevant groups: severely reduced LVEF (≥ 35%), mildly to moderately reduced LVEF (35–50%), and normal LVEF (≥ 50%). MR was classified as Mild (< 30ml), Moderate grade II (30–44ml), Moderate Grade III (45–59ml), and severe (> 60ml) for SW1, SW2, and echocardiography.

### 3. Artificial Intelligence Image Analysis (SW 2)

**Figure 3:**
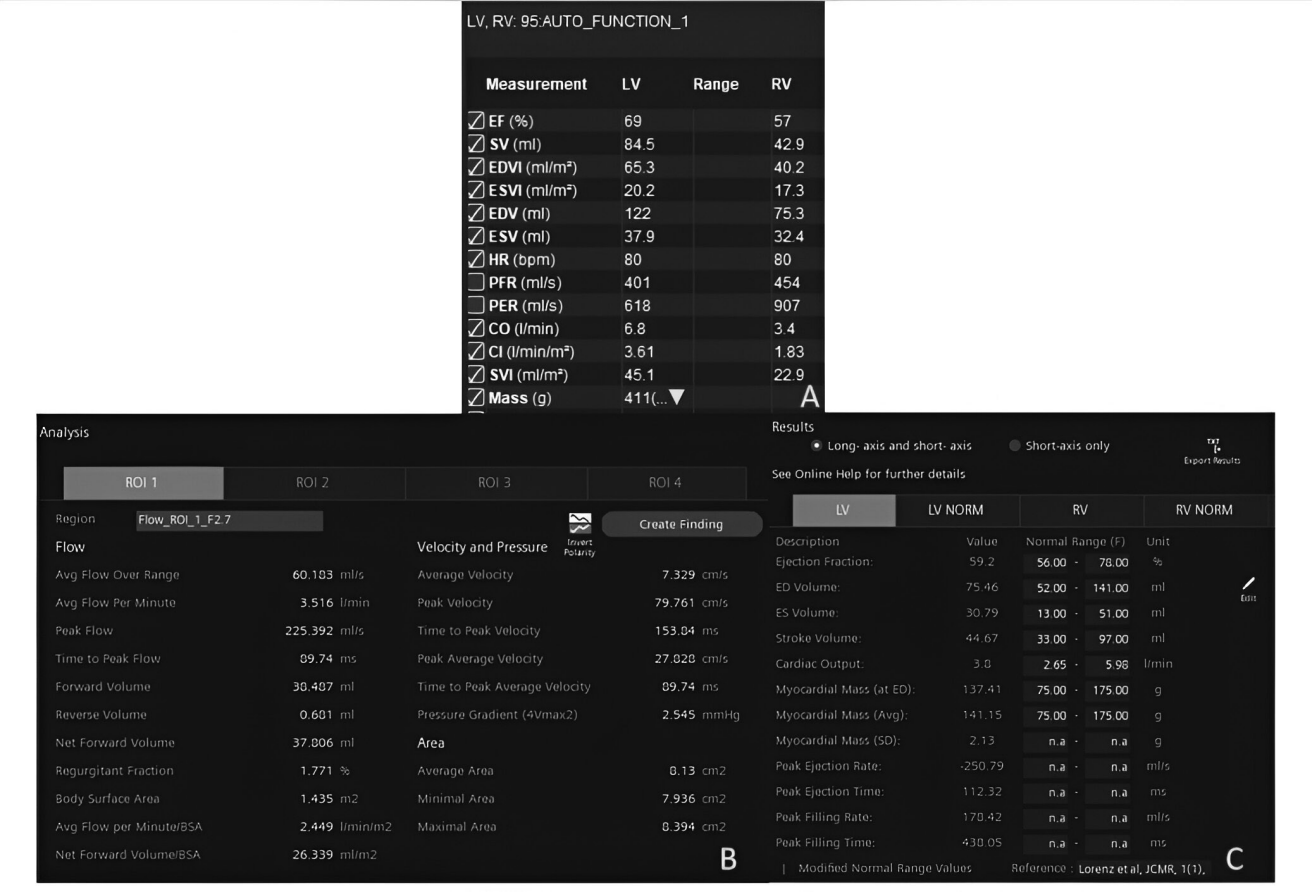
**A)** Post-processing output of left ventricular functional assessment using deep learning-based CMR software. The chart displays automatically calculated parameters including the ejection fraction (EF), stroke volume (SV), and end-diastolic volume (EDV), derived from cine short-axis images. Although the segmentation process is fully automated, only the quantitative results are shown here, highlighting the software’s ability to rapidly generate reproducible cardiac function metrics without manual contouring. **B)** Post-processing results of aortic flow quantification using manual segmentation in conventional CMR software. The image illustrates operator-defined contouring on velocity-encoded phase-contrast images to derive key hemodynamic parameters, including aortic forward volume, regurgitant (backward) volume, regurgitation fraction, peak systolic velocity, and pressure gradient. Unlike the automated approach, this method requires manual delineation of the aortic lumen in each phase, thus making the process time-consuming and operator-dependent but still considered the current reference standard for flow assessment. **C)** Post-processing results of aortic flow quantification using deep learning-based CMR software. The image displays automated analysis of velocity-encoded phase-contrast data, including the aortic forward volume, backward (regurgitant) volume, regurgitation fraction, peak systolic velocity, and pressure gradient across the aortic valve. The software automatically identifies the region of interest and performs contouring across all cardiac phases, enabling rapid, operator-independent assessment of aortic hemodynamics with high reproducibility.

The DL-EF, SV, EDV values were determined from cine short-axis images by using fully automated commercially available algorithms: SuiteHeart, Neosoft, Pewaukee, Wisconsin, USA. The vendor provided virtual training of how to optimally use the software. A single user was trained to use both software packages. Fully automated segmentation was then performed without any user input. The automatically and manually generated LVEDV, LVESV, AoF (Aortic forward flow), PG (Pressure gradient at LVOT) and LVEF were recorded (see Figure 3: A, B, C).

Aortic forward flow and regurgitation was calculated by using DL and the manual segmentation method. Analysis of pressure gradient at the level of LVOT was performed. MR was calculated by using the indirect method for both types of software (MR=LVSV-Aortic forward flow).

**Figure 4:**
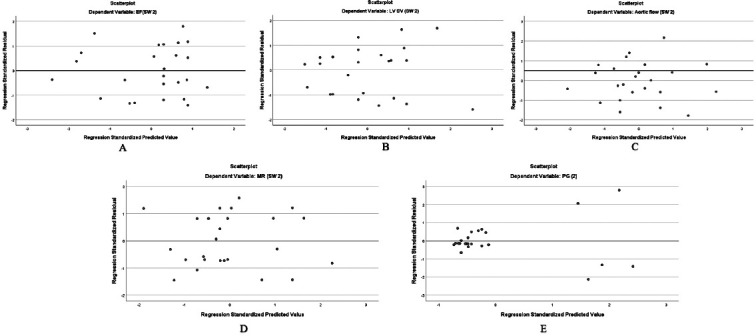
**A, B, C, D, E**. A- EF: R = 0.935, R^2^ = 0.873, F(1, 23) = 144.69, *p* < 0.001. The regression coefficient for Software 1 was 0.936 (*p* < 0.001), while the intercept was not statistically significant (B = 2.8, *p* = 0.553). B- LVSV: R = 0.97, R^2^ = 0.944, F(1, 23) = 185.04, *p* < 0.001. The coefficient for Software 1 was 0.984 (*p* = 0.001). C- AF: R = 0.91, R^2^ = 0.834, *p* < 0.001. The coefficient for Software 1 was 0.9 (*p* = 0.001). D- MR: R = 0.97, R^2^ = 0.95, *p* < 0.001. The coefficient for Software 1 was 0.9 (*p* = 0.001). E- PG: R = 0.99, R^2^ = 0.9, *p* < 0.001. The coefficient for Software 1 was 0.9 (*p* = 0.001).

**Figure 5:**
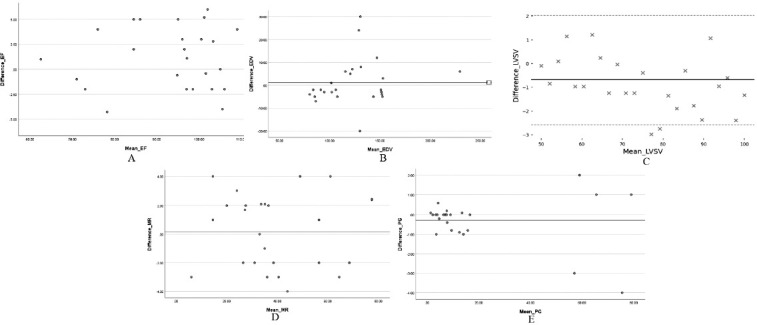
**A, B, C, D, E**. Bland–Altman plots showing the agreement between the two software tools for the quantification of (A) ejection fraction (EF), (B) left ventricular end-diastolic volume (LVEDV), (C) left ventricular stroke volume (LVSV), (D) mitral regurgitation (MR), and (E) pressure gradient (PG). The mean differences (bias) and limits of agreement (LOA) are displayed for each parameter. EF showed a mean difference of 2.1% with LOA from -5.9% to 10.1%. LVEDV had a mean difference of 1.2 mL and LOA from -18.4 to 20.8 mL. LVSV exhibited a negligible bias of -0.68 mL, with LOA from -2.58 to 2.03 mL. MR presented a mean difference of 0.17%, with LOA from -3.75% to 4.09%. PG demonstrated a mean difference of -0.28 mmHg and LOA from -2.58 to 2.03 mmHg. Across all parameters, the plots indicate good agreement without evidence of systematic or proportional bias, suggesting that the two software tools yield comparable measurements.

## Results

### Ejection Fraction (EF)

The mean EF values obtained by using Software 1 (manual segmentation) and Software 2 (deep learning-based automated segmentation) were 62.4 ± 8.52% and 61.3 ± 8.54%, respectively. A paired *t*-test indicated no statistically significant difference between the two methods (mean difference: 2.1% ± 1.1, *p* = 0.93).

### Left Ventricular End-Diastolic Volume (LVEDV)

The mean difference in LVEDV between Software 1 and Software 2 was 9.32 ± 24.0 mL. This difference was not statistically significant (*p* = 0.90).

### Left Ventricular Stroke Volume (LVSV)

The mean LVSV difference between the two software tools was -0.68 ± 3.91 mL, with no significant difference observed (*p* = 0.393, two-sided; *p* = 0.88, one-sided).

### Aortic Forward Flow (AF)

The mean difference in the aortic forward flow was 0.004 ± 4.89 mL, with a *p*-value of 0.91, indicating no significant discrepancy between the software outputs.

### Mitral Regurgitation (MR)

The mean difference in mitral regurgitation measurements was 0.004 ± 4.8%, and the difference was not statistically significant (*p* = 0.97).

### Pressure Gradient (PG)

The pressure gradient differed by -0.28 ± 1.1 mm Hg between Software 1 and Software 2, with no significant difference detected (*p* = 0.99).

Collectively, these findings demonstrate that deep learning-based segmentation yields comparable measurements to manual segmentation across all assessed cardiac parameters, with no statistically significant differences observed in EF, LVEDV, LVSV, AF, MR, or PG (see Table 2). This supports the clinical feasibility of using deep learning tools for automated cardiac function assessment in patients with *Hypertrophic CardioMyopathy* (HCM).

**Table 2:** Correlation analysis between automated and manual methods for key cardiac functional parameters. The table shows the correlation coefficient (R), coefficient of determination (R^2^), ANOVA *p*-value for significance testing, and the slope (coefficient) obtained from manual method comparison for each parameter. All correlations were statistically significant (*p* < 0.001).

S. No	Parameter	R	R^2^	Anova (p value)	Coefficient (Manual method)
1	Ejection fraction	0.935	0.873	< 0.001	0.8
2	End diastolic volume	0.92	0.81	< 0.001	0.84
3	Stroke volume	0.97	0.94	< 0.001	0.98
4	Aortic flow	0.91	0.83	< 0.001	0.9
5	Mitral regurgitation	0.92	0.88	< 0.001	0.88
6	Pressure Gradient	0.9	0.87	< 0.001	1.01

### Regression Analysis

Linear regression analysis demonstrated strong correlations between manual and deep learning-based measurements across all the evaluated parameters. The ejection fraction showed a high correlation (R = 0.93, R^2^ = 0.873, *p* < 0.001) with a slope of 0.936. The stroke volume exhibited excellent agreement (R = 0.97, R^2^ = 0.944, *p* < 0.001; slope = 0.984). The aortic forward flow also correlated strongly between the methods (R = 0.91, R^2^ = 0.834, *p* < 0.001; slope = 0.900), as did mitral regurgitation (R = 0.92, R^2^ = 0.880, *p* < 0.001; slope = 0.880). The pressure gradient across the LVOT demonstrated near-perfect alignment (R = 0.99, R^2^ = 0.873, *p* < 0.001) with a slope of 1.01, indicating close correspondence between the software outputs.

### Intraclass Correlation Coefficient (ICC)

The *Intraclass Correlation Coefficient* (ICC) analysis showed good to excellent agreement between manual and deep learning-based segmentation methods for all the measured parameters. The ejection fraction demonstrated an ICC of 0.935 (95% CI: 0.85–0.97), while LVEDV and LVSV showed ICCs of 0.89 (95% CI: 0.78–0.95) and 0.97 (95% CI: 0.93–0.98), respectively. The aortic forward flow and mitral regurgitation also exhibited strong reliability, with ICCs of 0.95 (95% CI: 0.92–0.97) and 0.96 (95% CI: 0.86–0.98). The pressure gradient across the LVOT yielded an ICC of 0.85 (95% CI: 0.76–0.94). All results were statistically significant (*p* < 0.001).

**Table 3:** Interclass correlation coefficients (ICC) were calculated to assess the agreement between automated and manual methods for each cardiac parameter. High ICC values (≥ 0.85) with statistical significance (*p* < 0.001) indicate excellent reliability

S. No	Parameter	n	Interclass correlation	Sig.
1	Ejection Fraction	25	.93	<.001
2	End Diastolic volume	25	.89	<.001
3	Stroke volume	25	.97	<.001
4	Aortic flow	25	.95	<.001
5	Mitral Regurgitation	25	.96	<.001
6	Pressure Gradient	25	.85	<.001

### Bland–Altman Analysis

Bland–Altman analysis demonstrated good agreement between manual and deep learning-based methods across all the evaluated parameters, with no evidence of systematic bias. For the ejection fraction, the mean difference was 2.1%, with *Limits Of Agreement* (LOA) ranging from -5.9% to 10.1%. The left ventricular end-diastolic volume showed a mean difference of 1.2 mL (LOA: -18.4 to 20.8 mL), while the stroke volume had a mean difference of -0.68 mL (LOA: -2.58 to 2.03 mL). Mitral regurgitation demonstrated close agreement, with a mean difference of 0.17% and LOA between -3.75% and 4.09%. The pressure gradient across the LVOT differed by -0.28 mmHg (LOA: -2.58 to 2.03 mmHg). Among the 19 patients with mild mitral regurgitation identified on echocardiography, cardiac MRI findings were concordant in most cases, although MRI indicated moderate MR in three patients.

**Table 4:** Bland–Altman analysis was performed to assess the agreement between automated and manual measurements. The table summarizes the minimum and maximum differences, mean bias, standard deviation (SD), and upper and lower limits of agreement (LOA, calculated as mean difference ± 1.96 × SD) for each parameter. Small mean differences and narrow LOA indicate strong agreement between the relevant datapoints.

S.No	Parameter	n	Minimum difference	Maximum difference	Mean difference	Std. Dev	Upper LOA	Lower LOA
1	Ejection fraction	25	-4.28	6.00	2.1	1.5	10.1	-5.9
2	EDV	25	-20	30	1.2	4.1	20.8	-18.4
3	Aortic flow	25	-10	9	.004	4.1	7.8	-7.6
4	Mitral Regurgitation	25	-4.0	4	.17	2.5	4	-3.7
5	Pressure Gradient	25	-4	2	-0.2	1.1	2.03	-2.58

### Analysis Time Comparison

The average time required for manual segmentation when using Software 1 was 30 ± 7 minutes, compared to 7 ± 3 minutes for the deep learning-based automated method (Software 2), thus reflecting a substantial time-saving advantage with AI-based analysis.

## Discussion

HCM presents unique challenges in imaging due to asymmetric hypertrophy, papillary muscle abnormalities, and *LV Outflow Tract* (LVOT) obstruction [6]. While echocardiography remains the initial imaging modality, *Cardiac Magnetic Resonance Imaging* (CMR) offers superior spatial resolution and reproducibility, which makes it the reference standard for quantifying cardiac parameters. However, manual segmentation is time-consuming and subject to inter- and intra-observer variability, which limits its widespread adoption, particularly in high-volume or resource-limited settings. The implementation of DL-based algorithms offers the potential to streamline the workflow, reduce the reporting times, and enhance the accessibility to advanced imaging techniques [14].

Several previous studies have demonstrated the accuracy and efficiency of *Deep Learning* (DL)-based segmentation in cardiac magnetic resonance imaging. Bai et al. implemented fully convolutional networks for large-scale automated CMR segmentation and reported high agreement with manual measurements, thus highlighting the potential of DL models in clinical workflows [15]. Similarly, Avendi et al. combined CNNs with deformable models for fully automatic LV segmentation and showed excellent performance in delineating endocardial and epicardial borders [16]. Wolterink et al. further validated DL models by using cine MR datasets for both segmentation and disease classification, thus achieving robust accuracy across multiple cardiac conditions [17]. Priya et al. demonstrated a strong correlation between automatically and manually derived cardiac volumes, indicating that the automatic segmentation method is highly accurate and consistent with expert manual delineation in cardiomyopathy [18]. However, these studies primarily focused on standard morphologies or generalized disease cohorts. The present study evaluated the equivalence of automated *Deep Learning* (DL)-based software and manual segmentation in the assessment of cardiac parameters, including *Left Ventricular Ejection Fraction* (LVEF), *Left Ventricular End-Diastolic Volume* (LVEDV), *Left Ventricular Stroke Volume* (LVSV), along with *Aortic Forward Flow* (AF), *Mitral Regurgitation* (MR), and *Pressure Gradient* (PG) at LVOT in patients with *Hypertrophic CardioMyopathy* (HCM) which, to the best of our knowledge, is not evaluated in literature yet.

The results demonstrate a high degree of agreement between the two methods, with no statistically significant differences in any of the measured parameters.

Excellent correlation was achieved with manual segmentation for the key parameters, such as LVEF (R = 0.935, R^2^ = 0.873) and LVEDV (R = 0.9, R^2^ = 0.811), which is crucial for clinical decision-making in HCM. The absence of any statistically significant differences (*p* > 0.05) between the two methods across all parameters suggests that DL algorithms can provide reliable quantitative assessments with minimal user intervention. This finding aligns with previous studies reporting the accuracy and efficiency of DL-based segmentation for cardiac imaging [19,20]. Linear regression showed strong agreement between the manual and deep learning-based methods across all cardiac parameters. The ejection fraction (R = 0.93, R^2^ = 0.873, slope = 0.936, *p* < 0.001) and stroke volume (R = 0.97, R^2^ = 0.944, slope = 0.984, *p* < 0.001) demonstrated excellent correlation. The aortic forward flow (R = 0.91, R^2^ = 0.834, slope = 0.900, *p* < 0.001) and mitral regurgitation (R = 0.92, R^2^ = 0.880, slope = 0.880, *p* < 0.001) also correlated well. The pressure gradient showed near-perfect concordance (R = 0.99, slope = 1.01, *p* < 0.001). These results confirm that DL-based segmentation yields measurements comparable to manual methods in HCM.

*Intraclass Correlation Coefficient* (ICC) analysis showed good to excellent agreement between manual and DL-based methods. EF had an ICC of 0.935 (95% CI: 0.85–0.97), LVEDV: 0.89 (0.78–0.95), and LVSV: 0.97 (0.93–0.98), all with *p* < 0.001. Similarly, AF and MR showed strong reliability with ICCs of 0.95 (0.92–0.97) and 0.96 (0.86–0.98), respectively. The pressure gradient also demonstrated acceptable agreement (ICC = 0.85, 95% CI: 0.76–0.94, *p* < 0.001). These findings further support the consistency of DL-based quantification in the clinical evaluation of HCM.

Bland–Altman analysis confirmed good agreement between the two methods, with no significant systematic bias across parameters. For EF, the mean difference was 2.1%, with the limits of agreement (LOA) from 5.9% to 10.1%. LVEDV showed a mean difference of 1.2 mL (LOA: -18.4 to 20.8 mL), whereas LVSV had a negligible bias of 0.68 mL (LOA: 2.58 to 2.03 mL). MR measurements showed minimal variation (mean difference 0.17%, LOA: -3.75% to 4.09%), while PG had a mean difference of 0.28 mmHg (LOA: 2.58 to 2.03 mmHg). These results indicate strong consistency between manual and DL-based measurements.

## Advantages and Limitations of DL in HCM Imaging

The DL-based approach offers several advantages, including reduced time for analysis and improved reproducibility. Automated segmentation eliminates the fatigue associated with manual analysis, thus allowing radiologists to focus on the clinical interpretation and patient care. Furthermore, by standardizing measurements, DL algorithms can facilitate multicentre studies and longitudinal monitoring of patients with HCM [20].

However, this study highlights specific challenges in applying DL algorithms to HCM imaging. The complex morphology of HCM, including asymmetric hypertrophy and LVOT obstruction, may pose difficulties for automated segmentation. Although the results showed no significant differences, the mean discrepancies observed in parameters such as LVEDV (9.32 ± 24 mL) and PG (-0.28 ± 1.1 mmHg) suggest that further refinement of DL algorithms is needed to account for the unique anatomical and functional characteristics of HCM. Additionally, the current DL-based software predominantly focuses on the aortic and pulmonary artery flow, with limited capability to measure LVOT pressure gradients which are critical for surgical planning and prognosis in HCM.

## Future Directions

The findings of this study underscore the need for HCM-specific optimization of DL algorithms. Future research should focus on integrating advanced AI techniques, such as hybrid models combining rule-based algorithms and deep learning, so that to further improve accuracy in measurements. Furthermore, the development of AI models trained on larger and more diverse datasets, including patients with varying phenotypes of HCM, may enhance the generalizability and robustness of automated segmentation.

This study demonstrated the equivalence of DL-based and manual methods, whereas the clinical impact of DL algorithms on workflow efficiency, diagnostic accuracy, and patient outcomes still warrant further investigation. Prospective studies comparing DL-based CMR analysis with echocardiography and other imaging modalities in large HCM cohorts will provide valuable insights into the role of AI in the clinical practice. The strength of this study is the direct comparison of DL-based and manual segmentation using a robust paired analysis in a cohort of patients with HCM, a population where accurate quantification of cardiac parameters is critical for management. The use of multiple regression analyses further validated the strong correlation between the two methods.

The lack of statistically significant differences between manual and DL-based methods across all measured parameters suggests that deep learning tools can reliably replicate expert-level quantification. This has important clinical implications, particularly in healthcare settings with limited radiology staffing or high patient volumes. Automated segmentation reduces post-processing time, thereby enabling faster decision making and providing a broader access to advanced cardiac MRI. Moreover, by minimizing the manual workload, DL allows radiologists to focus more on the clinical interpretation and integration with the patient history. However, certain HCM phenotypes – such as those with atypical wall thickening patterns, mid-cavity obstruction, apical aneurysms, or prominent papillary muscles – may pose challenges for fully automated models. Studies have reported the coexistence of HCM and non-compaction cardiomyopathy [21]. The cases we had were insufficient for analysis of such coexistence. DL-based automatic segmentation and contouring may be challenging in such cases. In such cases, manual verification and correction remain important to ensure the diagnostic accuracy. Future work incorporating phenotype-specific training datasets may help enhance the DL performance in these subgroups.

However, the study has several limitations. The sample size was relatively small (n = 25), and the analysis was performed at a single centre using specific software tools. The generalizability of the findings to other DL algorithms and clinical settings may be limited. The absence of inter- and intra-observer variability assessment for manual segmentation represents a methodological limitation. Given the operator-dependent nature of manual contouring, particularly in morphologically complex conditions like HCM, quantification of the observer variability would have provided a more rigorous benchmark against which to assess the reproducibility and clinical robustness of the deep learning-based approach.

## Conclusion

DL-based automated segmentation demonstrates high accuracy and reliability for the quantification of cardiac parameters in patients with HCM, with results comparable to manual segmentation. The time required by SW 2 for manual analysis was 30 ± 7 minutes, whereas the time required for SW 1 (DL based automated segmentation) was 7 ± 3 minutes. While the current algorithms show promise for streamlining clinical workflows and enhancing accessibility to CMR, further optimization is still needed to address the unique challenges of HCM imaging. Future studies should focus on validating these findings in larger, multicenter cohorts, and exploring the integration of DL algorithms into the clinical practice so that to improve the outcomes for patients with HCM.
